# Effects of an Atypical Antipsychotic, Zotepine, on Astroglial L-Glutamate Release through Hemichannels: Exploring the Mechanism of Mood-Stabilising Antipsychotic Actions and Antipsychotic-Induced Convulsion

**DOI:** 10.3390/ph14111116

**Published:** 2021-10-31

**Authors:** Kouji Fukuyama, Motohiro Okada

**Affiliations:** Department of Neuropsychiatry, Division of Neuroscience, Graduate School of Medicine, Mie University, Tsu 514-8507, Japan; k-fukuyama@clin.medic.mie-u.ac.jp

**Keywords:** astrocyte, hemichannel, connexin43, L-glutamate, convulsion, zotepine

## Abstract

Accumulating neuropsychopharmacological evidence has suggested that functional abnormalities of astroglial transmission and protein kinase B (Akt) contribute to the pathophysiology and/or pathomechanisms of several neuropsychiatric disorders, such as epilepsy, schizophrenia, affective disorders and antipsychotic-induced convulsions. Therefore, to explore the pathophysiology of mood-stabilising antipsychotics and the proconvulsive actions of atypical antipsychotics, the present study determined the effects of a mood-stabilising, atypical, antipsychotic agent, zotepine (ZTP), on astroglial L-glutamate release and the expression of connexin43 (Cx43) protein in cortical, primary, cultured astrocytes using ultra-high-pressure liquid chromatography and capillary immunoblotting systems. Both acute and subchronic administrations of therapeutically relevant concentrations of ZTP did not affect astroglial L-glutamate release or Cx43 expression in plasma membranes; however, chronic administration of a therapeutically relevant concentration of ZTP increased astroglial L-glutamate release and Cx43 expression in the plasma membrane. Subchronic administrations of a supratherapeutic concentration of ZTP enhanced astroglial L-glutamate release and Cx43 expression in the plasma membrane, whereas acute administration of a supratherapeutic concentration of ZTP enhanced astroglial L-glutamate release without affecting Cx43 expression. These stimulatory effects of ZTP on astroglial L-glutamate release through activated hemichannels and Cx43 trafficking to the astroglial plasma membrane were suppressed by the Akt inhibitor. These results suggest that ZTP enhances astroglial L-glutamate release in a concentration-dependent and time-dependent manner due to the enhanced function of astroglial hemichannels, probably via activation of Akt signalling. Therefore, the enhanced astroglial L-glutamatergic transmission induced by ZTP is, at least partially, involved in the mood-stabilising antipsychotic and proconvulsive actions of ZTP.

## 1. Introduction

Zotepine (ZTP), 2-[(8-chlorodibenzo[b,f]thiepin-10-yl)oxy]-N,N-dimethylethan-1-amine, is the first atypical antipsychotic drug to have been approved [1,2,3]. ZTP was originally developed and launched in Japan in 1982; however, the term “atypical antipsychotics” did not exist at its development. Based on its pharmacodynamic profile and clinical features, ZTP was later proposed to be classified as an “atypical antipsychotic” [2,3,4,5]. Indeed, the receptor-binding profile of ZTP displays antagonism of the dopamine D2 and serotonin 5-HT2A receptors, similar to other atypical antipsychotics [4,5]. ZTP is effective in managing positive and negative symptoms of schizophrenia with low extrapyramidal symptom liability [6,7,8,9]. Furthermore, several clinical studies have reported the effectiveness of ZTP in treating bipolar disorder [10,11,12,13]. A recent meta-analysis found that ZTP was one of the most effective antipsychotics [14]. In spite of its effectiveness, ZTP, along with clozapine and quetiapine, was evaluated as the antipsychotic with the highest risk of convulsions [15,16,17,18]. Clinical studies reported that ZTP dose-dependently increased slow wave in electroencephalograms [19], similar to clozapine [20,21,22]. Additionally, the peripheral adverse effects of ZTP, such as pneumonia and cardiotoxicity [23,24], are also known to increase the risk of lethality of clozapine [22,25,26]. Recently, it was proposed that the stimulatory effect of clozapine on connexin43 (Cx43), which has hemichannel activity via activation of protein kinase B (Akt) signalling, plays important roles in the pathomechanisms of convulsion, pneumonia and cardiotoxicity induced by clozapine [22]. Indeed, upregulation of Cx43 was observed in the acute stages of hypertrophic and dilated cardiomyopathies [27], as well as in T lymphocytes of noninfectious pulmonary inflammation [28]. Interestingly, hyperactivation of glutamatergic tripartite synaptic transmission via upregulation of astroglial Cx43 also plays an important role in the pathomechanisms of epilepsy [29]. Therefore, these previous preclinical findings suggested that hyperactivation of astroglial hemichannels possibly contributes to the pathophysiology of the adverse effects of ZTP.

Astrocytes play important roles in tripartite synaptic transmission, including exocytotic and nonexocytotic gliotransmitter release through the vesicle, hemichannels and, reversely transporters of astrocytes [22,30,31,32,33]. The astroglial hemichannel is one of the major molecules for nonexocytotic astroglial transmitter release. A hemichannel is formed by six connexin units [22,34] and contributes to chemical connections between intra- and extra-cellular spaces [30,31]. Cx43 is the most widely and predominantly expressed isoform of the connexin family in astrocyte, myocardial and pulmonary cells [34]. Under physiological conditions, astroglial hemichannels exhibit low opening probability [35,36,37], whereas pathological conditions, such as depolarisation, ischaemia, specific cation mobilisation and phosphorylation, generate persistent hemichannel opening, resulting in sustained astroglial nonexocytotic release of excitatory L-glutamate, D-serine, adenosine triphosphate, kynurenine metabolites and eicosanoids, which have a toxic effect on homeostasis systems [32]. Thus, until recently, astroglial hemichannels have been considered to be molecules that contribute to brain damage [37]. In contrast, recent neuropsychopharmacological studies have indicated the possibility that regulation of astroglial Cx43-containing hemichannels could be novel targets for several neuropsychiatric disorders, such as bipolar disorder, schizophrenia, epilepsy, autism spectrum disorder and their associated cognitive impairment [22,32,33].

In particular, recent studies demonstrated that the mood-stabilising antipsychotics clozapine and quetiapine rapidly enhanced Cx43 trafficking to the astroglial plasma membrane and activated Cx43-containing hemichannels, resulting in the increasing release of excitatory astroglial transmitters, such as L-glutamate [33,38,39]. Indeed, first-line antiseizure drugs, such as brivaracetam, carbamazepine, lacosamide and zonisamide, suppress the astroglial hemichannel’s function [40,41]. Taken together with the clinical findings, these recent pharmacological demonstrations have suggested that activation of the excitatory gliotransmitter release via activated astroglial Cx43-containing hemichannels plays important roles in antipsychotic-induced convulsions. On the other hand, serotonin transporter-inhibiting antidepressants chronically enhance Cx43 synthesis but suppress astroglial hemichannel activity [35,42]. Although it includes some contradictions, the discrepancy between antidepressants and mood-stabilising atypical antipsychotics suggests a possible hypothesis that activation and inhibition of the astroglial Cx43-containing hemichannels contribute to mood-stabilising (antimanic) and antidepressive actions, respectively [22,33].

Traditionally, tripartite synaptic transmission referred to the transmission of D-serine, L-glutamate and ATP between neurones and astrocytes, whereas the latest interpretation of tripartite synaptic transmission has been extended to other transmission systems, such as monoaminergic tripartite synaptic transmission [43], since the expression of several serotonin (5-HT) receptors, including serotonin 5-HT1A and 5-HT7 receptors, in the astrocytes has been revealed [42,44,45]. The concept of the participation of monoaminergic transmission in tripartite synaptic transmission provides an opportunity to expand the novel monoaminergic hypothesis, in which the functional modulation of astrocytes is involved in the pathophysiology of some neuropsychiatric disorders [33,46]. Based on this background, in order to clarify the mechanisms of the antipsychotic, antimanic and proconvulsive actions of ZTP, the present study determined the concentration-dependent effects of ZTP on astroglial L-glutamate release and the Cx43 protein expression of primary cultured cortical astrocytes using ultra-high-pressure liquid chromatography and capillary immunoblotting systems.

## 2. Results

Astrocytes were prepared using a protocol from our previous studies [40]. In particular, to determine the basal astroglial L-glutamate release and the L-glutamate release through activated astroglial hemichannels, cultured astrocytes were incubated in artificial cerebrospinal fluid (ACSF) and high K^+^ (100 mM) with Ca^2^^+^-free ACSF (HK-ACSF) for 20 min (HK-ACSF-evoked stimulation) [40]. The details of the experimental designs are explained in the “Materials and Methods” section.

### 2.1. Effects of ZTP on Astroglial l-Glutamate Release

#### 2.1.1. Concentration-Dependent Effects of Acute and Subchronic Administration of ZTP on Astroglial l-Glutamate Release

**Figure 1:**
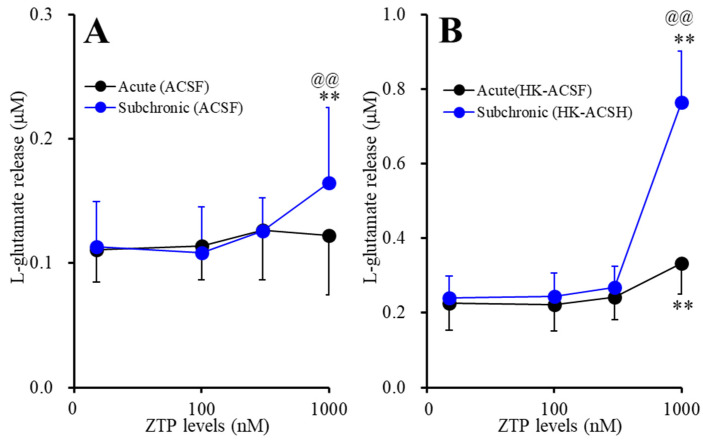
Concentration-dependent effects of the acute (black circles) and subchronic (blue circles) administrations of zotepine (ZTP: 100, 300 and 1000 nM) on the (**A**) basal and the (**B**) high (100 mM) K^+^ with Ca^2+^ free artificial cerebrospinal fluid (HK-ACSF)-evoked astroglial L-glutamate releases. Ordinate: mean ± SD (n = 6) of the astroglial L-glutamate release (μM), and abscissa: concentration of ZTP (nM). ** *p* < 0.01: relative to ZTP-free, @@ *p* < 0.01: relative to acute administration by multivariate analysis of variance (MANOVA) with Tukey’s (wholly significant difference) post hoc test.

Neither acute (120 min) nor subchronic (7 days) administration of therapeutically relevant concentrations of ZTP (100 and 300 nM) [47,48] affected basal astroglial L-glutamate release (Figure 1A,B). Acute administration of a supratherapeutic concentration of ZTP (1000 nM) [47,48] did not affect basal astroglial L-glutamate release, but subchronic administration of a supratherapeutic concentration of ZTP (1000 nM) slightly but significantly increased basal astroglial L-glutamate release (F_ZTP_(1.6, 16.3) = 10.6 (*p* < 0.01), F_administration_(1,10) = 0.2 (*p* > 0.1), F_ZTP*administration_(1.6, 16.3) = 5.6 (*p* < 0.05)) (Figure 1A).

Neither acute nor subchronic administration of therapeutically relevant concentrations of ZTP (100 and 300 nM) affected HK-ACSF-evoked astroglial L-glutamate release, whereas both acute and subchronic administration of a supratherapeutic concentration of ZTP (1000 nM) increased HK-ACSF-evoked astroglial L-glutamate release (F_ZTP_(1.2, 12.3) = 225.2 (*p* < 0.01), F_administration_(1,10) = 8.7 (*p* < 0.05), F_ZTP*administration_(1.2, 12.3) = 99.5 (*p* < 0.01)) (Figure 1B). The increase in HK-ACSF-evoked astroglial L-glutamate release induced by subchronic administration of a supratherapeutic concentration of ZTP was larger than that induced by acute administration of a supratherapeutic concentration of ZTP (Figure 1B).

#### 2.1.2. Interaction between Supratherapeutic Concentrations of ZTP and Astroglial Hemichannel Inhibitors on Astroglial l-Glutamate Release

To clarify the mechanisms of the stimulatory effects of supratherapeutic concentrations of ZTP on basal and HK-ACSF-evoked astroglial L-glutamate release, at DIV 28 after washout, during the pretreatment period, the cultured astrocytes were incubated in ACSF containing a supratherapeutic concentration of ZTP (1000 nM), with or without a selective Cx43 inhibitor, N-terminal transactivator of transcription GAP19 (TAT-GAP19: 10 μM), for 120 min [41]. After pretreatment, the astrocytes were incubated in ACSF (basal release) or HK-ACSF (HK-ACSF-evoked release) containing the same pretreatment agents for 20 min.

**Figure 2:**
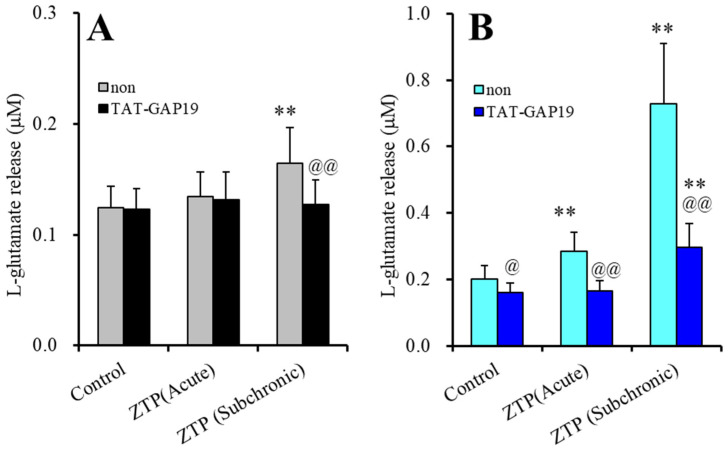
Effects of selective Cx43 inhibitor, N-terminal transactivator of transcription GAP19 (TAT-GAP19) (10 μM) on stimulatory effects of supratherapeutic concentration of ZTP (1000 nM) on basal (**A**) and HK-ACSF-evoked (**B**) astroglial L-glutamate releases. Ordinate: mean ± SD (n = 6) of astroglial L-glutamate release (μM). Light and dark colour columns indicate the incubation without (non) and with TAT-GAP19, respectively. ** *p* < 0.01: relative to non, @ *p* < 0.05, @@ *p* < 0.01 relative to control by MANOVA with Tukey’s (wholly significant difference) post-hoc test.

TAT-GAP19 did not affect the basal astroglial L-glutamate release, but the inhibited HK-ACSF-evoked L-glutamate release (Figure 2). Therefore, astroglial Cx43-containing hemichannels were not affected during the resting stage, whereas increasing the extracellular K^+^ level through Ca^2+^ removal activated astroglial Cx43-containing hemichannels. The stimulatory effects of subchronic administration of a supratherapeutic concentration of ZTP (1000 nM) on basal astroglial L-glutamate release was suppressed by TAT-GAP19 (F_ZTP_(2,20) = 18.2 (*p* < 0.01), F_TAT-GAP19_(1,10) = 1.2 (*p* > 0.1), F_ZTP*TAT-GAP19_(2,20) = 14.2 (*p* < 0.01)) (Figure 2A). TAT-GAP19 also suppressed the stimulatory effects of both acute and subchronic administrations of 1000 nM ZTP on HK-ACSF-evoked astroglial L-glutamate release (F_ZTP_(1.1, 10.9) = 108.7 (*p* < 0.01), F_TAT-GAP19_(1,10) = 20.4 (*p* < 0.01), F_ZTP*TAT-GAP19_(1.1, 10.9) = 34.2 (*p* < 0.01)) (Figure 2B).

These results indicated that astroglial hemichannels could not contribute to basal astroglial L-glutamate release during the resting stage, whereas Cx43-containing astroglial hemichannels were activated by HK-ACSF-evoked stimulation, resulting in enhanced astroglial L-glutamate release. Therefore, the supratherapeutic concentration of ZTP (1000 nM) enhanced astroglial L-glutamate release via activation of the function of Cx43-containing astroglial hemichannel under the both resting and hyperexcitable stages, since the selective Cx43 inhibitor, TAT-GAP19, prevented both increased basal and HK-ACSF-evoked L-glutamate release induced by a supratherapeutic concentration of ZTP.

#### 2.1.3. Interaction between Supratherapeutic Concentrations of ZTP and the Inhibitor of Protein Kinase B on Astroglial l-Glutamate Release

The stimulatory effects of subchronic administration of clozapine and quetiapine were mediated by enhanced Cx43 trafficking to the plasma membrane via activation of protein kinase B (Akt) activity [38]. Therefore, according to previous findings, to explore the mechanisms of the stimulatory effects of acute and subchronic administration of a supratherapeutic concentration of ZTP (1000 nM) on astroglial L-glutamate release through astroglial hemichannels, astrocytes were exposed to an AKT inhibitor, 10-[4’-(N,N-diethylamino)butyl]-2-chlorophenoxazine hydrochloride (DEBC: 10 μM), for 6 hr [38].

**Figure 3:**
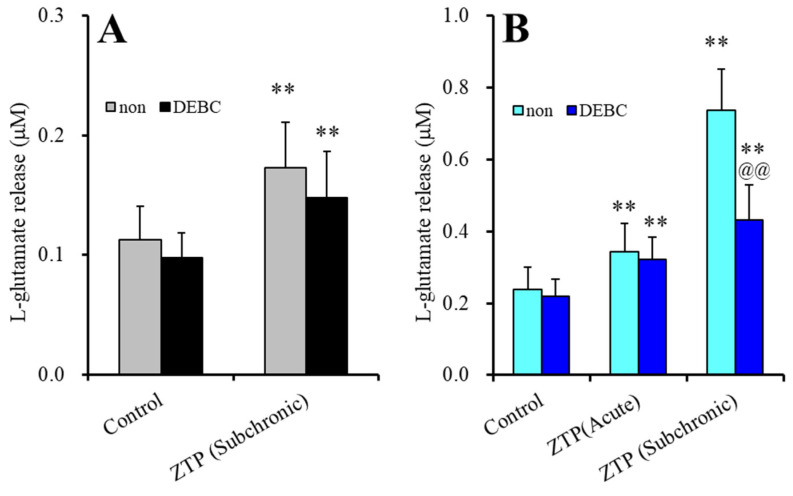
Effects of acute exposure to Akt inhibitor, 10-[4’-(N,N-diethylamino)butyl]-2-chlorophenoxazine hydrochloride (DEBC: 10 μM) on the stimulatory effects of supratherapeutic concentration of ZTP (1000 nM) on basal (**A**) and HK-ACSF-evoked (**B**) astroglial L-glutamate releases. Light and dark colour columns indicate the incubation without (non) and with DEBC, respectively. ** *p* < 0.01: relative to non, @@ *p* < 0.01 relative to control by MANOVA with Tukey’s (wholly significant difference) post-hoc test.

Akt inhibition induced by 10 μM DEBC for 6 hr did not affect either basal or HK-ACSF-evoked astroglial L-glutamate release (Figure 3). The stimulatory effects of subchronic administration of a supratherapeutic concentration of ZTP (1000 nM) on basal L-glutamate release were not affected by DEBC (Figure 3A). Therefore, the stimulatory effect of subchronic administration of a supratherapeutic concentration of ZTP on basal L-glutamate release was stimulated by activation of Cx43-containing hemichannels rather than Cx43 trafficking to the plasma membrane (possibly through direct activation of Cx43-containing hemichannel activity).

The stimulatory effects of acute administration of a supratherapeutic concentration of ZTP (1000 nM) on HK-ACSF-evoked L-glutamate release were also not affected by DEBC, whereas DEBC suppressed the HK-ACSF-evoked L-glutamate release induced by subchronic administration of a supratherapeutic concentration of ZTP (1000 nM) (F_ZTP_(2,20) = 190.1 (*p* < 0.01), F_TDBEC_(1,10) = 8.5 (*p* < 0.05), F_ZTP*DBEC_(2,20) = 34.0(*p* < 0.01)) (Figure 3B). These results suggest that the stimulatory effect of ZTP on HK-ACSF-evoked L-glutamate release is generated by subchronic activation of Cx43 trafficking to the plasma membrane, and acute direct activation of Cx43-containing hemichannel activity but not by trafficking.

### 2.2. Effects of ZTP on the Expression of Cx43 Protein in Astrocytes

We have already demonstrated that subchronic administration of several mood-stabilising atypical antipsychotics, namely clozapine, quetiapine and brexpiprazole, enhanced the trafficking of Cx43 to the astroglial plasma membrane without affecting Cx43 synthesis [38,39]. The trafficking of Cx43 to the plasma membrane is regulated by several intracellular phosphorylation signals [29,33,42]. Therefore, the concentration-dependent and time-dependent effects of ZTP (100, 300 and 1000 nM) on Cx43 expression in the plasma membrane and cytosol fractions were determined using a capillary immunoblotting system. The details of the experimental designs are explained in the “Materials and Methods” section.

#### 2.2.1. Concentration-Dependent Effects of Subchronic Administration of ZTP on Cx43 Expression in the Cytosol Fraction

**Figure 4:**
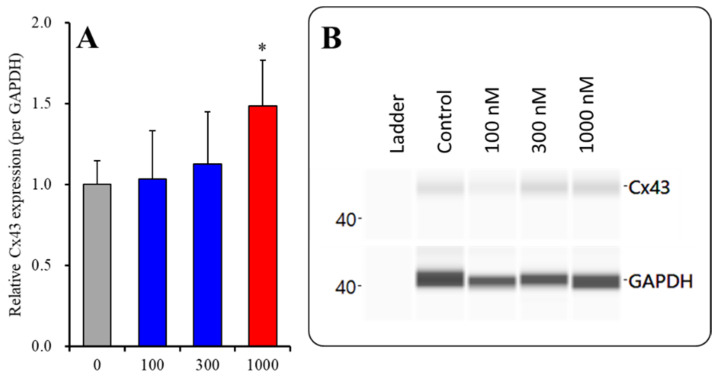
Concentration-dependent effects of subchronic administration of ZTP on Cx43 protein expression in the cytosol fraction (**A**) and their pseudogel images, using capillary immunoblotting (**B**). Ordinate: mean ± SD (n = 6) of the relative protein level of Cx43 (per GAPDH). Concentration-dependent effects of ZTP (100, 300 and 1000 nM) on Cx43 expression in the cytosol fraction of the primary cultured astrocytes were analysed by one-way ANOVA with Tukey’s (wholly significant difference) post hoc test (* *p* < 0.05 vs. ZTP free (0)).

**Figure 5:**
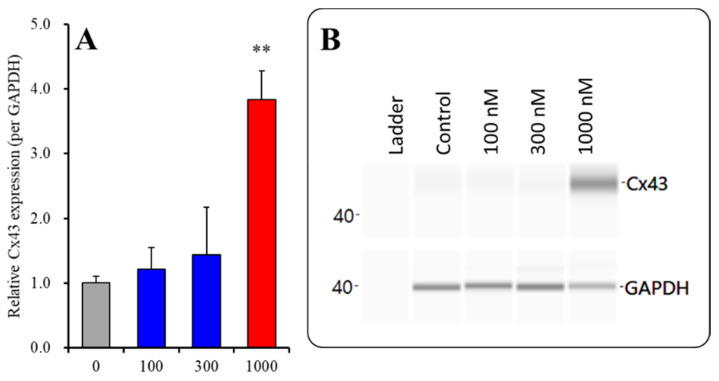
Concentration-dependent effects of subchronic administration of ZTP on Cx43 protein expression in the plasma membrane fraction (**A**) and their pseudogel images, using capillary immunoblotting (**B**). Ordinate: mean ± SD (n = 6) of the relative protein level of Cx43 (per GAPDH). Concentration-dependent effects of ZTP (100, 300 and 1000 nM) on Cx43 expression in the plasma membrane fraction of the primary cultured astrocytes were analysed by one-way ANOVA with Tukey’s (wholly significant difference) post hoc test (** *p* < 0.01 vs. ZTP free (0)).

To clarify the concentration-dependent effects of subchronic administration of ZTP on Cx43 protein expression levels in the astroglial cytosol and plasma membrane, astrocytes were incubated in fDMEM containing ZTP (100, 300 and 1000 nM) for 7 days (from DIV 21 to DIV 28). Subchronic administration of ZTP concentration-dependently increased the Cx43 level in the cytosol fraction (F(3,20) = 4.1 (*p* < 0.05)) (Figure 4). In particular, therapeutically relevant concentrations of ZTP (100 and 300 nM) did not affect Cx43 levels in the cytosol fraction, but the supratherapeutic concentration of ZTP (1000 nM) increased Cx43 protein expression levels in the cytosol fraction (Figure 4). Subchronic administration of ZTP concentration-dependently increased Cx43 protein expression levels in the plasma membrane fraction (F(3,20) = 49.1 (*p* < 0.01)) (Figure 5). Specifically, therapeutic concentrations of ZTP (100 and 300 nM) did not affect Cx43 levels in the plasma membrane fraction, but the supratherapeutic concentration of ZTP (1000 nM) increased Cx43 levels in the plasma membrane fraction (Figure 5).

Subchronic administration of a supratherapeutic concentration of ZTP increased Cx43 expression in both the cytosol and plasma membrane; however, the increase in the Cx43 level in the plasma membrane was twice that in cytosol. Therefore, subchronic administration of a supratherapeutic concentration of ZTP enhanced both Cx43 synthesis and Cx43 trafficking to the plasma membrane.

#### 2.2.2. Interaction between Subchronic Administration of a Supratherapeutic Concentration of ZTP and Acute Administration of an Akt Inhibitor on Cx43 Protein Expression in the Plasma Membrane Fraction

**Figure 6:**
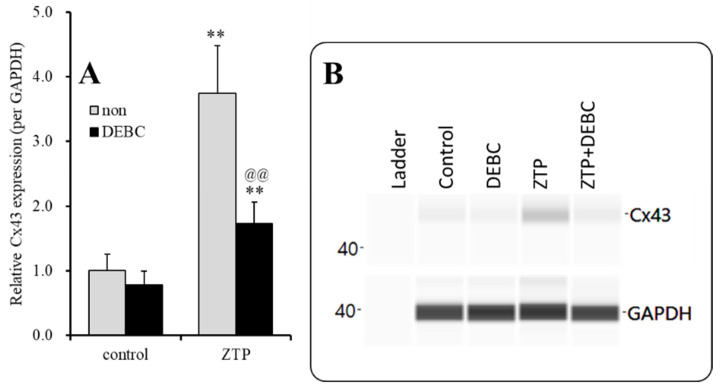
Interaction between subchronic administration of supratherapeutic concentration of ZTP and Akt inhibitor (DEBC) on Cx43 protein expression in the astroglial plasma membrane fraction (**A**) and their pseudogel images, using capillary immunoblotting (**B**). Ordinate: mean ± SD (n = 6) of the relative protein level of Cx43 (per GAPDH). Effects of ZTP (1000 nM) and Akt inhibitor (DEBC: 10 μM) on Cx43 expression in the plasma membrane fraction of the primary cultured astrocytes were analysed by two-way ANOVA with Tukey’s (wholly significant difference) post hoc test (** *p* < 0.01 vs. control, @@ *p* < 0.01 vs. non).

To clarify the effects of the ZTP-induced increase in Cx43 expression in the plasma membrane, the effects of an Akt inhibitor, 10 μM DEBC, on the increase in Cx43 expression in the plasma membrane induced by subchronic administration of a supratherapeutic concentration of ZTP (1000 nM) were determined. Before the pretreatment period, astrocytes were incubated in fDMEM containing without or with 10 μM DEBC for 6 hr [29]. DEBC did not affect Cx43 levels in the plasma membrane fraction, but inhibited the increase in Cx43 levels in the plasma membrane induced by subchronic administration of a supratherapeutic concentration of ZTP (1000 nM) (F_ZTP_(1,20)=29.7 (*p* < 0.01), F_DEBC_(1,20)=25.9 (*p* < 0.01), F_ZTP*DEBC_(1,20)=108.3 (*p* < 0.01)) (Figure 6).

### 2.3. Effects of Acute Administration of a Supratherapeutic Concentration of ZTP on Cx43 Expression in the Plasma Membrane Fraction and Chronic Administration of Therapeutically Relevant Concentrations of ZTP on Cx43 Expression in the Plasma Membrane Fraction and Astroglial L-Glutamate Release

#### 2.3.1. Effects of Acute Administration of Supratherapeutic Concentration of ZTP and Chronic Administration of Therapeutic-relevant Concentration of ZTP on Cx43 Expression in the Plasma Membrane Fraction

**Figure 7:**
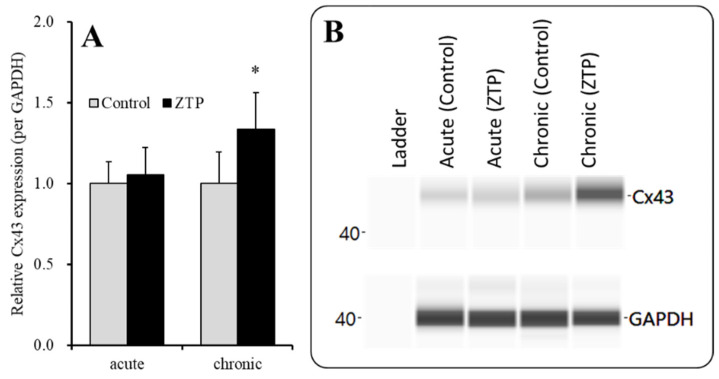
Effects of acute (120 min) administration of supratherapeutic concentration of ZTP (1000 nM) and chronic (14 days) administration of therapeutically relevant concentrations of ZTP (300 nM) on Cx43 protein expression in the plasma membrane fraction (**A**) and their pseudogel images, using capillary immunoblotting (**B**). Ordinate: mean ± SD (n = 6) of the relative protein level of Cx43 (per GAPDH). Effects of ZTP on Cx43 expression in the plasma membrane fraction of the primary cultured astrocytes were analysed by student T-test (* *p* < 0.05 vs. control: ZTP free).

To clarify the mechanisms of the stimulatory effects of acute administration of a supratherapeutic concentration of ZTP (1000 nM) on HK-ACSF-evoked astroglial L-glutamate release, at DIV 28, during the pretreatment period, astrocytes were incubated in the ACSF containing ZTP (1000 nM) for 120 min. Acute administration of a supratherapeutic concentration of ZTP (1000 nM) did not affect Cx43 levels in the plasma membrane fraction (Figure 7). To study the effects of chronic (14 days) administration of a therapeutically relevant concentration of ZTP (300 nM) on Cx43 protein expression in the plasma membrane, astrocytes were incubated in fDMEM containing ZTP (300 nM) for 14 days (from DIV 14 to DIV 28). Unlike with subchronic administration, chronic administration of therapeutically relevant concentrations of ZTP slightly but significantly increased Cx43 protein expression levels in the plasma membrane fraction (Figure 7).

#### 2.3.2. Effects of Chronic Administration of Therapeutically Relevant Concentrations of ZTP on Astroglial L-glutamate Release

**Figure 8:**
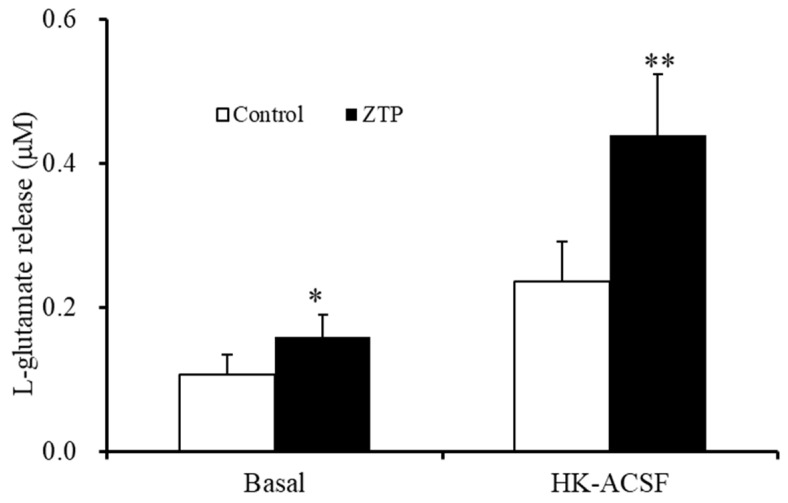
Effects of the chronic administrations of therapeutically relevant concentration of ZTP (300 nM) on basal and HK-ACSF-evoked astroglial L-glutamate releases. Ordinate: mean ± SD (n = 6) of the astroglial L-glutamate release (μM). * *p* < 0.05, ** *p* < 0.01: relative to ZTP-free (control) by student T-test.

To identify whether the increased Cx43 expression in the plasma membrane induced by chronic administration of a therapeutically relevant concentration of ZTP (300 nM) affected astroglial transmission, the effects of chronic (14 days) administration of a therapeutically relevant concentration of ZTP (300 nM) on basal and HK-ACSF-evoked astroglial L-glutamate release were determined. Subchronic (7 days) administration of a therapeutically relevant concentration of ZTP (300 nM) did not affect basal or HK-ACSF-evoked astroglial L-glutamate release (Figure 1), whereas chronic administration of 300 nM ZTP increased both basal and HK-ACSF-evoked astroglial L-glutamate release (Figure 8).

## 3. Discussion

### 3.1. Effects of ZTP on Astroglial L-Glutamate Release and Hemichannel Activity

The present study demonstrated that the effects of ZTP on astroglial transmission could be divided into concentration-dependent and time-dependent factors. The results of the present study are summarised in Table 1.

The supratherapeutic concentration of ZTP (1000 nM) could acutely (within 120 min of exposure) activate astroglial L-glutamate release through activated Cx43-containing hemichannels (Figure 1 and Table 1) without affecting Cx43 expression levels in the astroglial plasma membrane fraction (Figure 7 and Table 1). Thus, the acute stimulatory effects of the supratherapeutic concentration of ZTP on L-glutamate release was independent of the Cx43 expression level in the astroglial plasma membrane. These results indicate that the supratherapeutic concentration of ZTP directly enhanced the permeability of activated astroglial Cx43-containing hemichannels. The increase in Cx43 expression in the plasma membrane induced by the supratherapeutic concentration of ZTP required subchronic (7 days) administration (acute administration had no effect) (Figure 5 and Figure 7 and Table 1). Furthermore, an increase Cx43 expression in the plasma membrane induced by a supratherapeutic concentration of ZTP enhanced both basal astroglial L-glutamate release and L-glutamate release through activated Cx43-containing hemichannels. This enhanced astroglial L-glutamate release induced by subchronic administration of a supratherapeutic concentration of ZTP was suppressed by the selective Cx43 inhibitor TAT-GAP19 (Figure 2 and Table 1). Therefore, subchronic administration of a supratherapeutic concentration of ZTP enhanced Cx43-containing hemichannel activity qualitatively (enhancement of hemichannel permeability) and quantitatively (increasing functional hemichannels in the plasma membrane). The quantitative enhancement of astroglial hemichannels was probably generated by activation of Akt-dependent Cx43 trafficking to the plasma membrane, similar to the effects of clozapine and quetiapine [22,38,39], since the Akt inhibitor DEBC suppressed the enhancement of astroglial L-glutamate release and Cx43 expression in the plasma membrane induced by subchronic administration of a supratherapeutic concentration of ZTP (Figure 3, 6 and Table 1). Unlike the supratherapeutic concentration, neither acute (120 min) nor subchronic (7 days) administration of therapeutically relevant concentrations of ZTP (100 and 300 nM) affected astroglial L-glutamate release (Figure 1 and Table 1) or Cx43 expression in the plasma membrane (Figure 5 and Table 1). However, chronic administration (for 14 days) of a therapeutically relevant concentration of ZTP increased Cx43 expression in the plasma membrane (Figure 7 and Table 1) and astroglial L-glutamate release (basal L-glutamate release and L-glutamate release through activated Cx43-containing hemichannels) (Figure 8 and Table 1). The effects of subchronic administration of a supratherapeutic concentration of ZTP and chronic administration of a therapeutically relevant concentration of ZTP on quantitative Cx43 expression and qualitative hemichannel activity seemed to be generated by similar mechanisms, possibly activation of Akt signalling, similar to clozapine and quetiapine [38].

Earlier, we reported that systemic administration of ZTP dose-dependently increased the release of various transmitters, including L-glutamate, GABA, dopamine and norepinephrine, in the prefrontal cortex by using microdialysis [49]. Other atypical antipsychotics, such as aripiprazole, clozapine lurasidone, quetiapine and risperidone also dose-dependently increased the release of various neurotransmitters in the prefrontal cortex [22,33,50,51]. These preclinical findings indicated that the hypofrontality of patients with schizophrenia [52] is probably improved or compensated for by atypical antipsychotics; however, detailed mechanisms of the stimulatory effects of ZTP on L-glutamate release in the prefrontal cortex remain to be clarified [49]. Clinically, at least one or more than one week of intake duration was required to achieve the antipsychotic effects of ZTP [6,53]. Taken together with the clinical findings, the temporal coincidence between the stimulatory effects of therapeutically relevant concentrations of ZTP on astroglial L-glutamate release through Cx43-containing hemichannels and the onset of the clinical antipsychotic effects of ZTP indicates that the time-dependent stimulatory effects of therapeutically relevant concentrations of ZTP on astroglial L-glutamate release and Cx43 expression in the plasma membrane are probably involved in the clinical action of ZTP.

The present study could not identify the mechanisms of enhanced Akt signalling, which is a candidate intracellular signal for the increase in Cx43 trafficking to the plasma membrane induced by ZTP. Traditionally, several pharmacological studies revealed that inhibition of the dopamine D2 and serotonin 5-HT2A receptors disinhibited Akt signalling [54,55,56,57]. ZTP is a high-affinity antagonist of the dopamine D2 and serotonin 5-HT2A receptors [4,5]. The immunoreactivity of both the dopamine D2 and serotonin 5-HT2A receptors has been detected in rodent astrocytes [58,59]. Therefore, the antagonism of ZTP against dopamine D2 and serotonin 5-HT2A receptors probably contributes to the disinhibition of Akt signalling. It is well known that a number of atypical antipsychotics are antagonists of dopamine D2 and serotonin 5-HT2A receptors [2,3]. These findings provide us with the hypothesis that atypical antipsychotics possibly enhance astroglial hemichannel function via disinhibition of Akt signalling. The effects of atypical antipsychotics on Cx43 and Akt signalling in the astrocytes will be clarified in further studies.

### 3.2. A Candidate Mechanism of the Clinical Action of ZTP Associated with Astrocytes

It has been well known that some antipsychotics have a high risk of convulsions [15,16]. The incidence of clozapine-induced convulsion was approximately 1–7.5% [60,61]. The risk dosage of clozapine for convulsions was over 600 mg/kg/day, and the duration was 12 weeks [60]. A study on a large sample reported that the incidence of ZTP-induced convulsion was approximately 6~17% (7/115 and 22/129) [17,18]. The risk dosage of ZTP for convulsions was over 150~350 mg/kg/day and the average duration was 21~49 days [17,62,63]. The rapid titration of doses is also a risk factor of ZTP- and clozapine-induced convulsions [60,61]. Notably, the minimum convulsion risk dose of ZTP (350 mg/day) is within approved the therapeutic daily dose (150~450 mg/day) [17,62,63]. Furthermore, the average time of onset for convulsions of ZTP (7 weeks) [17,62,63] was faster than that of clozapine (12 weeks) [60,61]. Therefore, these previous clinical reports suggested that ZTP seems to be an atypical antipsychotic with a higher risk of convulsion compared with clozapine.

Recently, some of the first-line antiseizure drugs, brivaracetam, lacosamide and zonisamide, were found to suppress astroglial L-glutamate release or Cx43 trafficking to the plasma membrane, but carbamazepine had no effect [40,41]. Furthermore, upregulation of Cx43 in the focus region was observed in a rat model of autosomal dominant sleep-related hypermotor epilepsy [29,64,65], which is suppressed by zonisamide (zonisamide-sensitive) but is not affected by carbamazepine (carbamazepine resistance) [32,66]. Additionally, a number of preclinical studies have reported that hemichannel inhibitors have antiepileptic or antiseizure activities [37]. These preclinical findings suggest that the upregulated hyperactivation of astroglial Cx43-containing hemichannels plays an important roles in epileptogenesis/inctogenesis [32].

Based on the clinical and preclinical findings described above, the present study demonstrated a candidate mechanism of ZTP-induced convulsion associated with astroglial hemichannels. Acute administration (120 min) of a supratherapeutic concentration of ZTP enhanced astroglial L-glutamate release via direct activation of Cx43-containing hemichannel activity but did not affect Cx43 expression in the plasma membrane. Therapeutically relevant concentrations of ZTP had no effect acutely, but chronically enhanced astroglial L-glutamate release and Cx43 expression in the plasma membrane. These results suggest that the activation of astroglial Cx43-containing hemichannels is probably involved in the mechanisms of ZTP-induced convulsion.

Several post mortem studies have reported that expression of the mRNA or protein of Cx43 were downregulated in the locus coeruleus, frontal cortex and thalamus of patients with mood disorders [33,67,68,69,70,71]; however, an abnormality in glial size, which suggests the existence of hemichannel or gap–junction dysfunctions, was not observed in patients with schizophrenia [72,73]. Additionally, contrary to mood-stabilising atypical antipsychotics, serotonin transporter-inhibiting antidepressants enhance the synthesis of Cx43 (possibly through enhanced transcription of Cx43) but suppress the Cx43-containing hemichannels’ permeability [33,42,74,75,76,77]. Taken together with the effects of antidepressants, the present results suggest that inhibition and activation of astroglial hemichannels contribute to antidepressive and antimanic actions, respectively [33]. In other words, the functional abnormalities of astroglial hemichannels possibly contribute to the pathophysiology and pathomechanisms of mood disorders other than schizophrenia. The effectiveness of ZTP in acute mania has been established [12,13]. Moderate doses of ZTP (median daily dosage: 250 mg/day) displayed a rapid onset (within 1 week) of effectiveness for treatment of acute mania [12,13]. Therefore, the stimulatory effects of ZTP on astroglial L-glutamate release via direct activation of hemichannel permeability with an increase in Cx43 expression in the plasma membrane probably contribute to the antimanic action of ZTP, similar to other mood-stabilising atypical antipsychotics, such as clozapine and quetiapine [38,39].

## 4. Materials and Methods

### 4.1. Chemical Agents

Zotepine (ZTP), selective Cx43 inhibitor, N-terminal transactivator of transcription GAP19 (TAT-GAP19) and Akt inhibitor, 10-[4’-(N,N-diethylamino)butyl]-2-chlorophenoxazine hydrochloride (DEBC) were obtained from Funakoshi (Tokyo, Japan). All compounds were prepared on the day of the experiment. TAT-GAP19 and DEBC were dissolved in ACSF or fDMEM directly. ZTP was initially dissolved in dimethyl sulfoxide at 50 mM. The final dimethyl sulfoxide concentration was lower than 0.1% (*v*/*v*).

### 4.2. Preparation of Primary Astrocyte Culture

All animal care and experimental procedures described in this report complied with the Ethical Guidelines established by the Institutional Animal Care and Use Committee at Mie University, Japan (No. 2019-3) and are reported in accordance with the Animal Research: Reporting of In Vivo Experiments (ARRIVE) guidelines. Astrocytes were prepared using a protocol adapted from previously described methods.

Pregnant Sprague Dawley rats (SLC, Sizuoka, Japan) were housed individually in cages and kept in air-conditioned rooms (temperature, 22 ± 2 °C) set at a 12 h light/dark cycle, with free access to food and water. Cultured astrocytes were prepared from cortical astrocyte cultures of neonatal Sprague-Dawley rats (N = 54) sacrificed by decapitation at 0–24 h of age. The cerebral hemispheres were removed under dissecting microscope. Tissue was chopped into fine pieces using scissors and then triturated briefly with micropipette. Suspension was filtered using 70 µm nylon mesh (BD, Franklin Lakes, NJ, USA) and centrifuged. Pellets were then resuspended in 10 mL fDMEM, which was repeated three times. After DIV14, contaminating cells were removed by shaking in a standard incubator for 16 h at 200 rpm. Additionally, astrocytes were removed from flasks by trypsinization and seeded directly onto translucent polyethylene terephthalate (PET) membrane (1.0 μm) with 24-well plates (BD) at a density of 100 cells/cm^2^ for experiments from DIV14 to DIV28, the culture medium (fDMEM) was changed twice a week, and ZTP (100, 300 and 1000 nM) were added for subchronic administrations (for 14 days). On DIV28, cultured astrocytes were washed out using ACSF, and this was repeated three times [40].

The ACSF comprised NaCl 150.0 mM, KCl 3.0 mM, CaCl_2_ 1.4 mM, MgCl_2_ 0.8 mM, and glucose 5.5 mM, buffered to pH 7.3 with 20 mM HEPES buffer [64]. After the washout, astrocytes were incubated in ACSF (100 μL translucent PET membrane) containing ZTP (100, 300 and 1000 nM) at 35 °C for 60 min in a CO_2_ incubator (pretreatment incubation). After the pretreatment, astrocytes were then incubated in ACSF and 100 mM K^+^ with Ca^2^^+^-free (HK-ACSF) containing the same agents of pretreatment (20 min), followed by collection of the ACSF or HK-ACSF for analysis [64]. Each 100 μL of collected ACSF or HK-ACSF was filtered by Vivaspin 500-3K (Sartorius, Goerringen, Germany) and freeze-dried for storage at −80 °C until needed for analyses.

After the sampling of astroglial transmitter releases, plasma membrane proteins of cultured astrocytes were extracted using Minute Plasma Membrane Protein Isolation Kit (Invent Biotechnologies, Plymouth, MN, USA). Plasma membrane fractions were solubilised by radio immunoprecipitation assay buffer (Fujifilm-Wako, Osaka, Japan) containing protease inhibitor cocktail (Nacalai Tesque, Kyoto, Japan).

### 4.3. Ultra-High-Performance Liquid Chromatography (UHPLC)

L-glutamate levels were determined by using UHPLC equipped with xLC3185PU (Jasco, Tokyo, Japan) and fluorescence detection (xLC3120FP, Jasco) following dual derivatisation with isobutyryl-L-cysteine/o-phthalaldehyde. The derivatized samples (5 μL aliquots) were injected via an autosampler (xLC3059AS, Jasco). The analytical column (YMC Triat C18, particle 1.8 μm, 50 × 2.1 mm, YMC, Kyoto, Japan) was maintained at 45 °C, and the flow rate was set to 500 μL/min. A linear gradient elution program was used over a period of 10 min with mobile phases A (0.05 M citrate buffer, pH 5.0) and B (0.05 M citrate buffer containing 30% acetonitrile and 30% methanol, pH 3.5). The excitation/emission wavelengths of the fluorescence detector were set to 280/455 nm [51].

### 4.4. Capillary Immunoblotting Analysis

The capillary immunoblotting analysis was performed, using Wes (ProteinSimple, Santa Clara, CA, USA), according to the ProteinSimple user manual. The lysates of the primary cultured astrocytes were mixed with a master mix (ProteinSimple) to a final concentration of 1× sample buffer, 1× fluorescent molecular weight marker, and 40 mM dithiothreitol and then heated at 95 °C for 5 min. The samples, blocking reagents, primary antibodies, HRP-conjugated secondary antibodies, chemiluminescent substrate (SuperSignal West Femto: Thermo Fisher Scientific, Waltham, MA, USA), and separation and stacking matrices were also dispensed to the designated wells in a 25-well plate. After plate loading, the separation electrophoresis and immunodetection steps took place in the capillary system and were fully automated. A capillary immunoblotting analysis was carried out at room temperature, and the instrument’s default settings were used. Capillaries were first filled with a separation matrix followed by a stacking matrix, with about 40 nL of the sample used for loading. During electrophoresis, the proteins were separated by molecular weight through the stacking and separation matrices at 250 volts for 40–50 min and then immobilized on the capillary wall, using proprietary photoactivated capture chemistry. The matrices were then washed out. The capillaries were next incubated with a blocking reagent for 15 min, and the target proteins were immunoprobed with primary antibodies followed by HRP-conjugated secondary antibodies (Anti-Rabbit IgG HRP, A00098, 10 μg/mL, GenScript, Piscataway, NJ, USA). The antibodies of GAPDH (NB300-322, 1:100, Novus Biologicals, Littleton, CO, USA) and Cx43 (C6219, 1:100, Sigma-Aldrich, St. Louis, MO, USA) were diluted in an antibody diluent (ProteinSimple) [66].

### 4.5. Data Analysis

All statistical analyses complied with the recommendations on experimental design and analysis in pharmacology [78]. All experiments in this study were designed with equally sized animal groups (n = 6), without carrying out a formal power analysis, in keeping with previous studies. All values are expressed as the mean ± SD, and *p* < 0.05 (two-tailed) was considered statistically significant for all tests. Drug levels in acute and subchronic administrations were selected based on values in previous studies. Where possible, we sought to randomize and blind the data. In particular, for the determination of transmitter levels and protein expression, the sample order on the autosampler and Wes were determined by a random number table.

Concentration-dependent effects of acute and subchronic administrations of ZTP on basal astroglial L-glutamate release levels were analysed by multivariate analysis of variance (MANOVA) using BellCurve for Excel ver. 3.2 (Social Survey Research Information Co., Ltd., Tokyo, Japan). When the data did not violate the assumption of sphericity (*p* > 0.05), the F-value of the MANOVA was analysed, using sphericity-assumed degrees of freedom [78]. However, if the assumption of sphericity was violated (*p* < 0.05), the F-value was analysed, using Chi-Muller’s corrected degrees of freedom. When the F-value of MANOVA was significant, the data were analysed by a Tukey’s (wholly significant difference) multiple comparison test. Effects of chronic administration of a therapeutically relevant concentration of ZTP (300 nM) on astroglial L-glutamate release was analysed by Student’s T-test.

The concentration-dependent effects of ZTP on Cx43 protein levels in the cytosol plasma membrane fraction were analysed by one-way analysis of variance (ANOVA) with Tukey’s (wholly significant difference) multiple comparison, using BellCurve for Excel. The interaction between subchronic administration of ZTP and acute administration of Akt inhibitor on Cx43 protein levels in the plasma membrane fraction was analysed by two-way analysis of variance (ANOVA) with Tukey’s (wholly significant difference) multiple comparison, using BellCurve for Excel.

## 5. Conclusions

The present study determined the concentration- and time-dependent effects of ZTP on astroglial L-glutamate release and Cx43 protein expression in cultured astrocytes, to explore the mechanisms of the mood-stabilising antipsychotic and proconvulsive actions of ZTP. Neither acute (120 min) nor subchronic (7 days) administration of therapeutically relevant concentrations of ZTP affected astroglial L-glutamate release and Cx43 protein expression in the astroglial cytosol and plasma membrane; however, chronic (14 days) administration of therapeutically relevant concentrations of ZTP enhanced basal L-glutamate release and L-glutamate release through activated astroglial Cx43-containing hemichannels. Unlike therapeutically relevant concentrations, a supratherapeutic concentration of ZTP acutely increased astroglial L-glutamate release and subchronically increased Cx43 protein expression in the cytosol and plasma membrane. These stimulatory effects of ZTP on astroglial L-glutamate release and Cx43 expression in the plasma membrane were inhibited by an Akt inhibitor. These results suggest that ZTP enhanced astroglial glutamatergic transmission though increased trafficking of Cx43 to the plasma membrane via activation of Akt signalling, similar to clozapine and quetiapine; however, these effects of ZTP were more clearly expressed earlier at therapeutically relevant concentrations compared with clozapine and quetiapine. Therefore, the Akt-dependent activation of astroglial hemichannel activity by ZTP can explain the pathophysiology of ZTP’s mood-stabilising action and the possible mechanisms of several adverse effects (convulsions, cardiotoxicity and pneumonia) induced by ZTP.

## Figures and Tables

**Table 1:** Summary of the effects of zotepine (ZTP) on astroglial L-glutamate release and Cx43 expression in the astrocyte.

	Therapeutic-Relevant	Supratherapeutic	Therapeutic-Relevant	Supratherapeutic	
Release	Basal	(ACSF)	Activated	(HK-ACSF)	
Acute	Non	Increase GAP19(-)	Non	Increase GAP19(+) DEBC(-)	Figure 1, Figure 2 and Figure 3
Subchronic	Non	Increase, GAP19(+) DEBC (-)	Non	Increase GAP19(+) DEBC(+)	Figure 1, Figure 2 and Figure 3
Chronic	Increase		Increase		Figure 8
Cx43 Expression	Cytosol		Plasma membrane		
Acute				Non	Figure 7
Subchronic	Non	Increase	Non	Increase DEBC (+)	Figure 4, Figure 5 and Figure 6
Chronic			Increase		Figure 7

Cx43: connexin43, ACSF: artificial cerebrospinal fluid, HK-ACSF: incubated high (100 mM) K+ with Ca2+-free ACSF, GAP19: N-terminal transactivator of transcription GAP19 (selective Cx43 inhibitor), 10-[4′-(N,N-diethylamino)butyl]-2-chlorophenoxazine hydrochloride (protein kinase B inhibitor), non: no effect, increase: enhanced L-glutamate release or Cx43 expression, GAP19 (−): GAP19 insensitive, GAP19 (+): GAP19 sensitive, DEBC (−): DEBC insensitive, DEBC (+): DEBC sensitive.

## Data Availability

The data presented in this study are available on request from the corresponding author. The data are not publicly available due to equipment dependent data.
